# Polymorphisms of *CYP51A1* from Cholesterol Synthesis: Associations with Birth Weight and Maternal Lipid Levels and Impact on CYP51 Protein Structure

**DOI:** 10.1371/journal.pone.0082554

**Published:** 2013-12-17

**Authors:** Monika Lewińska, Urska Zelenko, Franci Merzel, Simona Golic Grdadolnik, Jeffrey C. Murray, Damjana Rozman

**Affiliations:** 1 Center for Functional Genomics and Bio-Chips, Faculty of Medicine, University of Ljubljana, Ljubljana, Slovenia; 2 Laboratory of Biomolecular Structure, National Institute of Chemistry Slovenia, Ljubljana, Slovenia; 3 EN-FIST Centre of Excellence, Ljubljana, Slovenia; 4 Department of Pediatrics, University of Iowa, Iowa City, Iowa, United States of America; Instituto de Tecnologica Química e Biológica, UNL, Portugal

## Abstract

We investigated the housekeeping cytochrome P450 *CYP51A1* encoding lanosterol 14α-demethylase from cholesterol synthesis that was so far not directly linked to human disorders. By direct sequencing of *CYP51A1* in 188 women with spontaneous preterm delivery and 188 unrelated preterm infants (gestational age <37 weeks) we identified 22 variants where 10 are novel and rare. In infants there were two novel *CYP51A1* variants where damaging effects of p.Tyr145Asp from the substrate recognition region, but not p.Asn193Asp, were predicted by PolyPhen2 and SIFT. This was confirmed by molecular modeling showing that Tyr145Asp substitution results in changed electrostatic potential of the CYP51 protein surface and lengthened distance to the heme which prevents hydrogen bonding. The CYP51 Tyr145Asp mutation is rare and thus very interesting for further structure/function relationship studies. From the 12 identified known variants rs6465348 was chosen for family based association studies due to its high minor allele frequency. Interestingly, this *CYP51A1* common variant associates with small for gestational age weight in newborns (p = 0.028) and lower blood total cholesterol and low density lipoprotein cholesterol levels in mothers in 2nd trimester of pregnancy (p = 0.042 and p = 0.046 respectively). Our results indicate a new link between a cholesterol synthesis gene *CYP51A1* and pregnancy pathologies.

## Introduction

The World Health Organization (WHO) defines preterm delivery (PTD) as birth before completed 37 weeks of gestation. It affects annually almost 15 million children worldwide, presenting the second largest direct cause of death in children younger than 5 years old [1], [2]. Both birth timing and birth weight are very important factors that may increase the risk of short-term medical complications (visual and hearing impairment, chronic lung disease) as well as long-term complications in adults, including learning disabilities and increased risk for cardiac and stroke related deaths [3], [4], [5]. Spontaneous PTD is a common and multifactorial disease in which life style and nutrition state interact with environmental, genetic and epigenetic factors. Additionally, it has been shown that genetic variations of both mother and fetus can contribute to PTD and low birth weight (LBW) [6], [7], [8].

Cholesterol is a crucial molecule for embryonic development and maintaining pregnancy [9]. In humans, it originates from *de novo* synthesis, dietary intake and in the case of the fetus also from maternal cholesterol transported *via* placenta [10]. The level of maternal blood lipids is important for pregnancy, physiological fetal development and also confers risk of PTD and LBW [11], [12]. Further importance of cholesterol synthesis and metabolism is illustrated in embryonic lethality or congenital defects due to mutations in genes of cholesterol synthesis and metabolism [13], [14], [15], cholesterol transport and the bile acids synthesis [16], [17].

**Figure 1:**
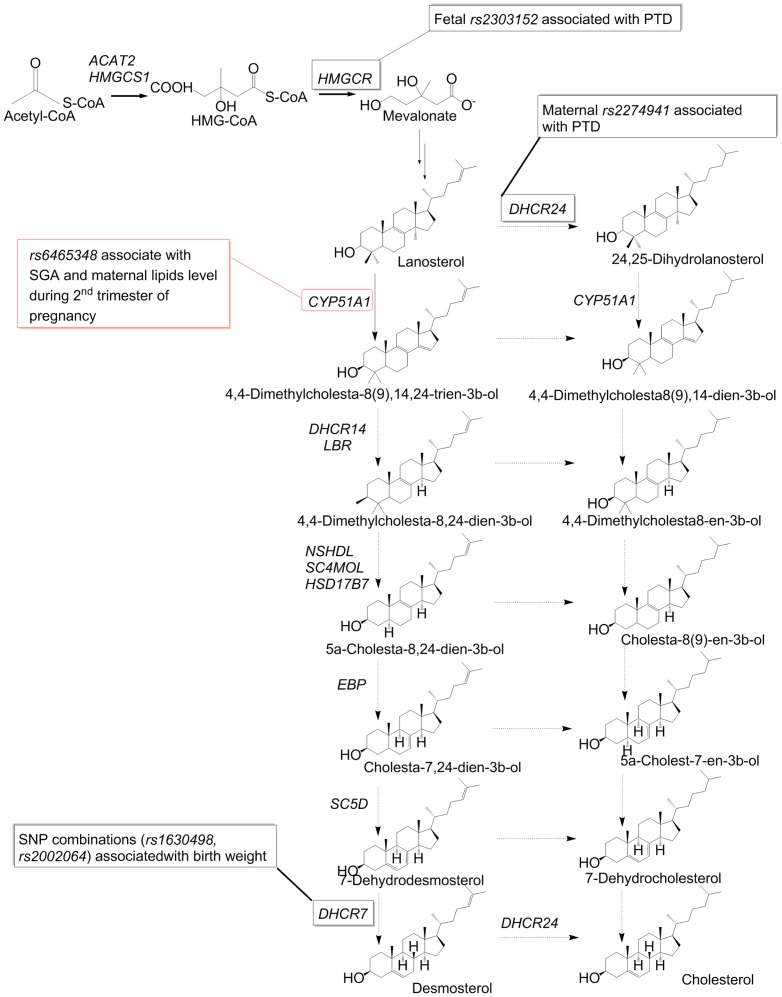
Cholesterol biosynthesis pathway with genes associated with preterm delivery and low birth weight according to Steffen at all [7] and with input from our study.

Cholesterol is synthetized in a multi-step pathway in all nucleated cells of the body. The regulatory enzyme of the late part is lanosterol 14α-demethylase (CYP51) that lies between HMGCR and DHCR7 or DHCR24 enzymes (Figure 1). CYP51 is the most evolutionarily-conserved member of the cytochrome P450 superfamily [18], [19], [20]. It catalyzes a complex 14α-demethylation reaction with the aid of cytochrome P450 reductase (POR) [21]. In humans, CYP51 converts lanosterol to FF-MAS (4,4-dimethyl-5alpha-cholesta-8,14,24-triene-3beta-ol) [22] and 24,25-dihydrolanosterol to dihydro-FF-MAS.

The *Cyp51* knockout mouse model shows embryonic lethality at day 15 post coitum with features similar to Antley-Bixler syndrome [23]. Since the identity of mouse and human CYP51 proteins is 91%, the mouse knockout phenotype would suggest embryonic lethality also in humans with two mutated *CYP51A1* alleles. Indeed, *CYP51A1* was so far not associated with human pathologies, but was found hemizygously deleted in a family affected by cerebral cavernous malformations [24] and proposed as a candidate gene for pediatric cataract [25].

In this study we tested the hypothesis that the heterozygous *CYP51A1* states might associate with cholesterol related pathologies that would present early in life. We investigated coding variants of *CYP51A1* in cohorts of neonates and/or mothers in pregnancies that resulted in preterm birth or growth retardation. The relevance of newly discovered *CYP51A1* functional polymorphisms was further explored by molecular modeling.

## Materials and Methods

### Study populations

**Figure 2:**
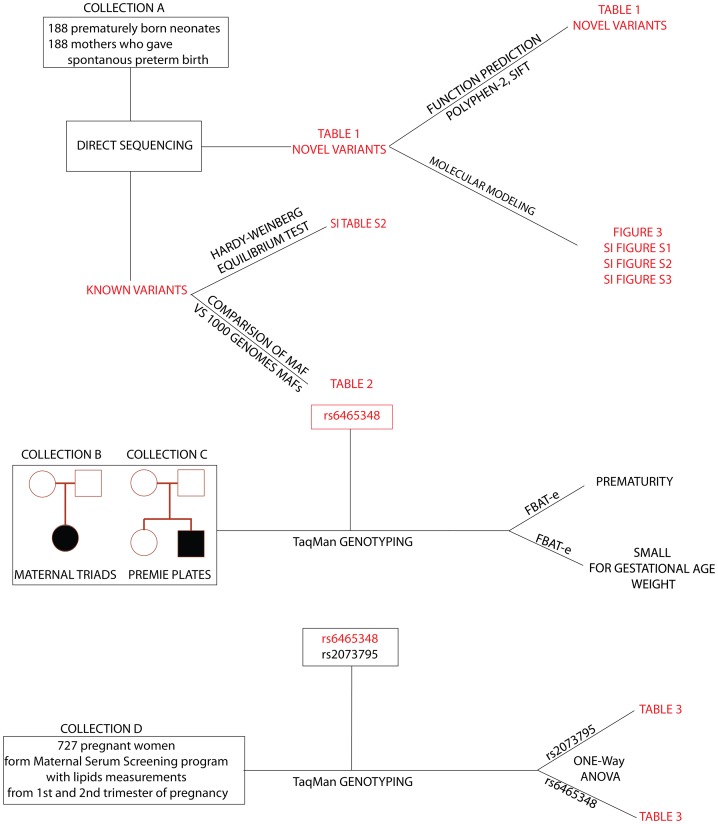
Experimental design and workflow. Panels show *CYP51A1* analysis workflow by direct sequencing (searching for novel functional variants) and the analysis on population level by genotyping of common*CYP51A1* variants and family based studies.

Informed, written consent was obtained for all participants. Study was approved by University of Iowa IRB-01 (Biomedical) Institutional Review Board (IRB number 200506792). All studied populations are graphically presented and the experimental design are shown in Fig. 2.

### Population screening of novel and common variants in CYP51A1 (collection A)

- 188 women of Western European descent who had a spontaneous preterm delivery and 188 unrelated preterm infants. Preterm birth (PTB) was defined as a live birth before 37 completed weeks of gestation based on ultrasound and last menstrual period, with ultrasound taking precedence if there was more than two weeks discrepancy. The exclusion criteria for mothers included medically indicated PTD (in women whose labor was induced or who were given cesarean sections before the onset of labor or premature rupture of membranes) and multiple pregnancies. The study included only neonates from singleton pregnancies, 94 of each gender.

### Genotyping of common CYP51A1 variants for association with PTD and SGA

The rs6465348 *CYP51A1* polymorphism has been chosen for genotyping in collections B and C, followed by family-based testing for allelic association between haplotype and prematurity.

- Maternal triads, where the ‘case’ was considered a mother who have gave preterm birth and at least one of her parents (948 nuclear families);
- Infant triads where a case was considered a prematurely born neonate (<37 weeks gestation) and at least one of its parents, excluding twins and infants with congenital anomalies (834 nuclear families);

The SGA/IUGR (Intrauterine growth restriction) was based on infant’s weight at birth at any gestational age being below the 10% for weight. We excluded from analyses infants with known causes of IUGR, including chromosome abnormalities, twins, maternal infection, severe insulin dependent diabetes etc.

### Genotyping of common CYP51A1 variant for association with blood lipids levels

The rs6465348 and rs2073795 *CYP51A1* polymorphisms have been chosen for genotyping collection D.

- Maternal serum plates, with collection of DNA from mothers drawn from the Iowa Maternal Serum Screening program, with available data of the total cholesterol (TC), triglycerides (TG), LDL cholesterol (LDL-c), HDL cholesterol (HDL-c) levels during the first and second trimesters of pregnancy (727 women).

### Searching for novel functional variants by sequencing

Genomic DNA was extracted from whole blood or saliva by standard methods. PCR amplicons covered were generated for the *CYP51A1* coding regions and intron-exon borders with 3’-UTR and 5’-UTR. PCR primers (Table S1) were designed using the Primer 3 Web site (http://frodo.wi.mit.edu). Sequencing was performed with the DNA sequencing kit, Big Dye™ Terminator Cycle Sequencing (Applied Biosystems) and reactions were resolved on an ABI Prism 3700 analyzer (Applied Biosystems), analyzed by Consed (http://www.genome.washington.edu/UWGC/analysistools/consed.cfm) and manually inspected to confirm possible mutations. For amplicons with potentially damaging mutation we performed also the reverse sequencing of the mutation-carring individual and his parents and further sequencing of additional 1000 premature neonates. The dbSNP (http://www.ncbi.nlm.nih.gov/SNP/), 1000Genomes (http://www.1000genomes.org/) and Exome Variant Server (EVS) (http://evs.gs.washington.edu/EVS/) were investigated to check if variants were previously reported. Known variants were tested for deviations from Hardy-Weinberg Equilibrium and for deviation of MAF from 1000 Genomes using exact probability test.

Functional mutations of *CYP51A1* gene (Table 1) have been analyzed with PolyPhen2 (http://genetics.bwh.harvard.edu/pph2/) and with SIFT (http://sift.bii.a-star.edu.sg/) prediction software and by molecular dynamics simulations carried out in CHARMM [26].

**Table 1:** Novel polymorphisms identified in *CYP51A1* with sequencing approach and PolyPhen 2 and SIFT predictions.

	Nucleotide change	Reference number in dbSNP (rs#)	Amino Acid substitution NP_000777.1/UniProt Q16850^a^	PolyPhen2 prediction (score)	SIFT prediction (score)
mothers	NM_000786.3:c.-255G>A	rs312262907			
	NM_000786.3:c.-225_-223delAGA	rs312262908			
	NM_000786.3:c.13G>A	rs312262909	p.Ala5Thr/-		
	NM_000786.3:c.*1121C>T	rs312262910			
infants	NM_000786.3:c.-37G>C	rs312262911			
	NM_000786.3:c.451T>G	rs312262912	p.Tyr151Asp/Tyr145Asp	Probably Damaging (0.999)	Damaging (0.00)
	NM_000786.3:c.595A>G	rs312262913	p.Asn199Asp/Asn193Asp	Benign (0.008)	Tolerated (1.00)
	NM_000786.3:c.595+112A>G	rs312262914			
	NM_000786.3:c.*228A>G	rs312262915			
	NM_000786.3:c.*944A>C	rs312262916			

^a^ Amino acid residue numbering in RefSeq database NP_000777.1 and in UniProt database Q16850;

### Genotyping common CYP51A1 variants by TaqMan assays

We have performed TaqMan genotyping of common *CYP51A1* variants by Applied Biosystems (Foster City, CA, USA) with pre-designed or validated SNP genotyping assays from Applied Biosystems under recommended conditions. SNP rs6465348 located in *CYP51A1* 3’ untranslated region was selected for genotyping in collections **B, C** and **D,** based on high minor allele frequency and the haplotype block structure of the *CYP51A1* gene. Additionally, rs2073795 located in intron 1 was genotyped in collection **D**, to improve the coverage of the locus.

### Statistical analyses

For known variants detected with sequencing we calculated minor allele frequencies separately for mothers and neonates and performed test of Hardy-Weinberg equilibrium using chi-square goodness-of-fit test to discover potential deviations from Hardy-Weinberg equilibrium. Due to the small sample size we evaluated results also by Freeman-Halton [27] extension of the Fisher exact probability test [28] (Fisher's Exact Test Calculator for a 2×3 Contingency Table, http://www.danielsoper.com/statcalc). Results are presented in Table S2.

To validate whether the known variants occur with same frequencies in collection A (separately for mothers and neonates) as in the general population, we applied the exact binominal test of goodness-of-fit [29]. We used expected proportions of alleles for general population calculated from MAF reported in dbSNP database that originates from the 1000 Genomes Project (http://www.ncbi.nlm.nih.gov/SNP/). Results are presented in Table 2. Calculations were performed using the spreadsheet available at http://udel.edu/~mcdonald/statexactbin.html that calculates two-tailed p-value using the method of small p-value.

**Table 2:** Minor allele frequencies and P-value of the exact binomial test of goodness-of-fit comparing MAF of known *CYP51A1* variants in neonates or mothers to the general population (1000 Genomes Project). Variants have been identified by sequencing.

rs number	MAF dbSNP	MAF neonates	p-value neonates	MAF mothers	p-value mothers
rs117814311	0.008	0.015	0.206	0.008	0.765
rs189739058	0.001	0.003	0.288	0.000	1.000
rs142544033	0.001	0.000	1.000	0.003	0.298
rs147205401	0.003	0.006	0.289	0.014	0.011
rs57218044	0.032	0.003	0.000*	0.000	0.000*
rs184213287	0.002	0.003	0.301	0.000	1.000
rs59683852	0.005	0.003	1.000	0.000	0.422
rs7797834	0.355	0.418	0.017*	0.360	0.895
rs7793861	0.358	0.413	0.033*	0.429	0.423
rs6465348	0.353	0.416	0.018*	0.420	0.019*
rs12673910	0.180	0.119	0.002*	0.061	0.000*
rs1135217	0.358	0.384	0.318	0.363	0.863

*P-value <0.05.

Analyzing the data in collections **B** and **C,** we took advantage of the samples available (neonate and parents) to use the family based allelic association test (FBAT). This approach is a case-only approach and relies on transmission distortion of parental alleles to measure effect and does not require controls (the non-transmitted alleles from the parents serve as the controls). The original transmission test for linkage disequilibrium TDT [30] uses only affected (binary trait) individuals, ignoring unaffected ones and non-informative families, where parents are homozygous. Due to generalizations (FBAT-e approach we were able to deal with gestational age and birth weight as continuous traits and take into account missing parents.

For collection **D** we applied one-way ANOVA using SPSS Statistics 19 software to calculate whether the lipid levels in pregnant women differs significantly according to the *CYP51A1* genotype.

### Molecular Modeling of novel functional variants

We performed molecular modeling of two of novel functional variants (Tyr145Asp and Asn193Asp) discovered with sequencing in neonates. Docking of lanosterol into the active site of human CYP51 structure 3LD6 [31], taken from RCSB Protein Data Bank was performed using the Glide (Glide, version 5.7, Schrödinger, LCC, New York, NY, 2011). The lanosterol conformation with the minimal GlideScore scoring function and the proper orientation of C30 atom relative to the heme iron was chosen for molecular dynamics simulation. The mutants Tyr145Asp and Asn193Asp (Q16850) were generated from the initial wild-type protein structure through the corresponding replacement of the residues Tyr145 and Asn193 by Asp. Each protein was solvated in explicit water environment modeled by the TIP3p water model [32]. The sodium and chloride ions were added to achieve electro-neutrality of the systems at physiological concentration. The all-atom simulation was carried out using program package CHARMM [26] and the available force field [33]. The force-field parameterization for lanosterol was taken from Cournia [34]. In addition, the proper connection between the heme (Fe) and Cys449 (S) atom was achieved by the correct protonation state of Cys449 and an additional harmonic restraint. Tetrahedral simulation cell with dimensions 76.3 Å and 75.6 Å was used together with periodic boundary conditions. Molecular dynamics (MD) of 5 ns equilibration period was followed by a 10 ns production run of each system. The MD simulations were run at constant pressure of 1 bar and temperature 300 K with the time step of 1 fs. The non-bonded interactions were treated using a set of cutoffs. The distance cutoff in generating the list of pairs was set to 13 Å. At 12 Å the switching function eliminated all contributions to the overall energy from pairwise interactions. At 10 Å the smoothing function began to reduce a pair's contribution. The simulated systems were visualized using Visual Molecular Dynamics (VMD) [35].

Existence of a hydrogen bond between two sites was determined according to the geometry criterion: a hydrogen bond is assumed to exist when two candidate atoms are closer than 2.4 Å and if the angle formed by donor-H-acceptor is greater than 150 degrees.

In order to visualize the electrostatic potential on the solvent accessible protein surface we have used Adaptive Poisson-Boltzmann Solver (APBS) program [36]. A range of electrostatic potential values between -5 to 5 kcal/mol is displayed.

### How polymorphic is CYP51A1?

Analysis of nucleotide variability of *CYP51A1* in comparison to other genes of cholesterol synthesis and to other cytochrome 450 genes as an out-group has been reported previously [37]. The analysis of missense mutations of *CYP51A1* and their influence on protein structure was evaluated with PolyPhen2 and SIFT (Table S3).

## Results

### *CYP51A1* in premature infants and unrelated mothers that gave birth prematurely - Summary of sequencing results

The human *CYP51A1* lies on Chromosome 7 q21.2. It has two processed pseudogenes at chromosomes 3 and 13 [38], [39]. To avoid amplification of *CYP51* pseudogenes that are both intronless, we designed primers in introns to amplify across the intron-exon borders of the functional *CYP51A1.* We sequenced the ten coding exons, intron-exon borders, 3’ and 5’ UTR regions in 188 white women who had spontaneous premature labor and 188 unrelated, preterm infants of Western European descent (Collection A, Figure 2). We identified 22 *CYP51A1* sequence variants, where 10 represented novel, rare, heterozygous variants (Table 1). Chi square goodness-of-fit test shows no deviations from Hardy-Weinberg equilibrium for known variants. Freeman-Halton extension of the Fisher exact probability test that is used for small populations also showed that none of the variants reached statistical significance (Table S2). The genotype distribution of known variants of *CYP51A1* in affected individuals (Collection A) does not deviate from HWE and does not suggest association of the locus with prematurity.

We also investigated whether frequencies of minor alleles (MAF) of known *CYP51A1* SNPs in neonates and mothers differ from the general population. The common variant rs6465348 was successfully sequenced in 172 premature babies (MAF 0.416) and 144 mothers (MAF 0.420). The exact binomial test of goodness-of-fit (Table 2) shows statistically significant difference between rs6465348 in both mothers who gave preterm birth (p = 0.019) and premature babies (p = 0.018) compared to the general population (1000 Genomes Project). Additionally, two other variants showed different frequencies in both mothers and neonates (rs57218044 and rs12673910), and two variants with significantly different frequencies in neonates only (rs7797834 and rs7793861). All *CYP51A1* SNPs reported in HapMap form one block and are thus predicted to be inherited together. In such cases choosing a single SNP with high MAF may be sufficient for association studies with the phenotype [40]. SNP rs6465348 from the *CYP51A1* 3’UTR exhibits a high MAF of 0.400 (HapMap Data Rel 24/phase II) and shows statistically significant differences in frequencies between mothers/neonates and 1000 Genomes. It was thus a good candidate SNP for further association studies.

### Genotyping of *CYP51A1* common variant rs6465348 in larger populations

To investigate the association between *CYP51A1* and prematurity in larger populations, we performed TaqMan genotyping of *rs6465348* followed by transmission test for linkage disequilibrium in two larger populations, where families of prematurely born babies and mothers who have preterm births (maternal triads **B.** and ‘premie’ plates **C.**). In maternal triads that contained 203 informative families rs6465348 did not associate with the phenotype (p = 0.913). In the collection of premature babies and their families (356 informative families) rs6465348 also fails to show significant association with prematurity (p = 0.880). These genotyping results and family-based approach show lack of association between common *CYP51A1* variants and prematurity which confirms the findings from Hardy-Weinberg Equilibrium test (Table S2).

The rs6465348 was analyzed further in association with small body weight for gestational age (SGA) as a phenotype. This phenotype was assigned by physicians in the newborns’ charts. The FBAT-e analysis shows moderate association between SGA and major allele (T) of rs6465348 reaching p = 0.028 in 27 informative families that did not include twin pregnancies. The association between this *CYP51* common variant and SGA phenotype needs to be further analyzed in a larger cohort.

The rs6465348 was investigated also for association with blood lipid levels in pregnant women. Here we applied as control SNP from intron 1 (rs2073795) with a lower MAF (0.178) compared to rs6465348 (0.400). In a study of 727 females, one way ANOVA showed that women homozygous for the recessive CC variant in rs6465348 have statistically significantly lower LDL cholesterol and total cholesterol (P = 0.046 and P = 0.042 respectively) in second trimester of pregnancy compared to women that had the TT variant. The SNP rs2073795 did not associate with blood lipids during pregnancy possibly due to lower power. P-values for both SNPs are presented in Table 3.

**Table 3:** One-Way ANOVA testing for association between listed parameters and SNP genotypes.

	One-Way ANOVA P-value	
Parameter	rs2073795	rs6465348
Total Cholesterol 1^st^ trimester	0.286	0.091
HDL Cholesterol 1^st^ trimester	0.379	0.617
LDL Cholesterol 1^st^ trimester	0.367	0.203
Triglycerides	0.897	0.470
Total Cholesterol 2^nd^ trimester	0.356	0.042*
HDL Cholesterol 2^nd^ trimester	0.338	0.745
LDL Cholesterol 2^nd^ trimester	0.499	0.046*
Triglycerides 2^nd^ trimester	0.852	0.319
Δ Total Cholesterol	0.367	0.307
ΔHDL	0.152	0.064
ΔLDL	0.716	0.908
Δ Triglycerides	0.605	0.438

*P-value <0.05.

### Evaluation of novel *CYP51A1* variants

Novel *CYP51A1* variants differ between mothers and unrelated preterm babies. In mothers the 4 novel variants consist of 1 deletion in 5’UTR, 2 SNPs in 3’UTR and 1 non-synonymous variant p.Ala5Thr in CYP51 (NP_000777.1). In neonates we identified 6 novel variants: 1 in 5’UTR, 2 in 3’UTR, 1 in exon-intron border and 2 non-synonymous variants, where Asn193Asp was predicted as tolerated and Tyr145Asp (UniProt Q16850) as probably damaging/damaging with PolyPhen2/SIFT.

The polymorphism c.451 T>G results in the non-synonymous substitution of Tyr145 (Uniprot Q16850) with aspartic acid. In the RefSeq database a longer CYP51 protein is included and this residue is numbered 151 (NP_000777.1 Tyr151). For protein structure we will use the Uniprot numbering in this report. This tyrosine is located in substrate recognition site 1 (SRS1) of the CYP51 protein [41]. Since both Polyphen2 and SIFT predict that this mutation might be damaging, we investigated how frequent it is in the population and what is the impact of this substitution on protein structure with molecular modeling. The amplicon including the variant in exon 3 c.451 T>G (p.Tyr145Asp) was sequenced in parents of the p.Tyr145Asp infant and in further 1000 premature neonates. The variant has been found in infant’s mother, but not in the additional 1000 cases. This indicates very low frequency of this polymorphism with potentially damaging effects on the CYP51 enzyme activity.

Functional relevance of novel *CYP51A1* variants – the molecular modeling approach

Even though the novel functional variants appear to be rare with currently estimated little contribution to PTD, they remain interesting in respect to protein function. Mutation c.13C>T leads to substitution of alanine (NP_000777.1) to threonine in the N-terminal domain responsible for membrane binding of CYP51 [31]. Substitution of a non-polar amino acid with a hydrophilic one may influence folding and binding capacity of the protein. The prediction of functional effects of this substitution with Polyphen-2 software was impossible and SIFT predicts substitution to be damaging, but with low confidence. Since the human CYP51 crystal structure lacks the N-terminal part of the protein [31], we could not predict the effect of this amino acid change by molecular modeling.

**Figure 3:**
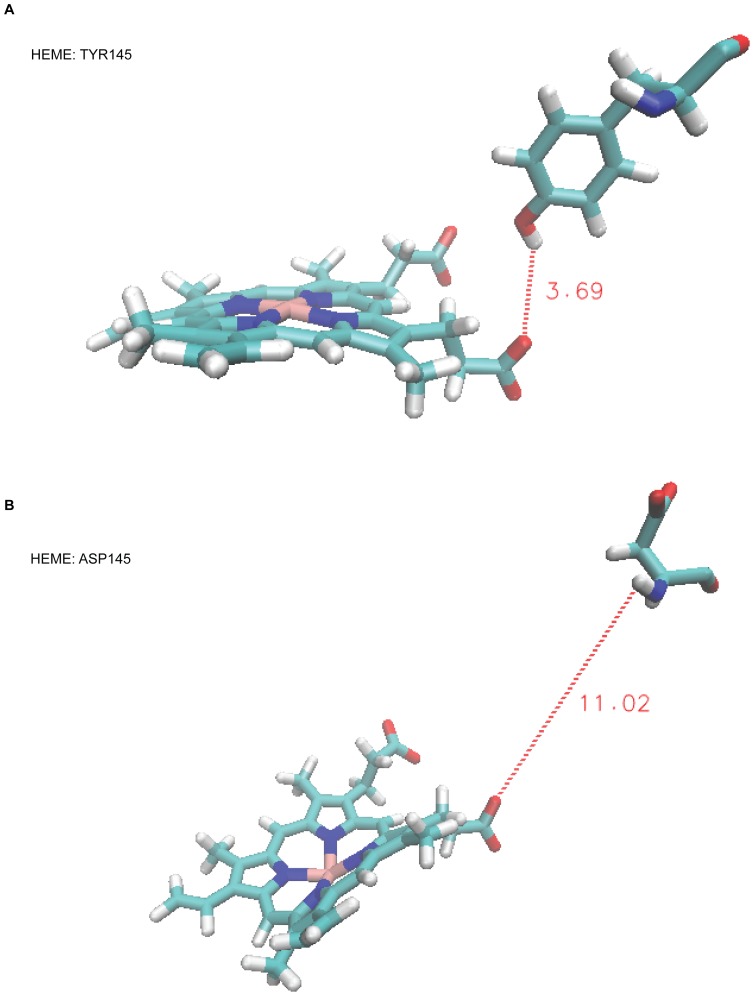
Relative positions of Tyr145 and Asp145 with respect to the heme. Dashed lines correspond to the temporary distances in Å between the heme oxygen and the corresponding hydrogen atoms which undergo time variations during the simulation. Occasionally, Tyr145 forms a hydrogen bond to the heme when internal motion of the protein brings Tyr145 closer to the heme. There is no hydrogen bond formed between Asp145 and heme as they are too far apart from each other.

To explore further the Polyphen2 and SIFT predictions of *CYP51A1* rare variants (p.Tyr145Asp and p.Asn193Asp) we applied molecular modeling. All-atom trajectories were used for characterization of the interaction between CYP51 and lanosterol inserted into the active site as shown in Figure S1. We investigated interaction energies between the substrate lanosterol and CYP51 wild-type and mutants (Figure S2A), distances between heme iron and C30 atom of lanosterol (Figure S2B), hydrogen bonding with heme (Figure 3) as well as electrostatic potential surface of the protein (Figure S3). Lanosterol was docked into the active site of human CYP51 (PDB 3LD6) producing 10 different poses (Table S4) using the software Glide. The three poses with the lowest GlideScore values showing very little variation in the molecule’s position as demonstrated in Figure S1 were selected for further structural optimization within the binding site. All three structures converged to the same conformation during the process of energy minimization in which the constraints used to impose initial rigidity of the binding site were gradually switched off. The minimized structure being characterized by the distance between the C30 atom and the heme iron 4,5 Å was further subjected to two point-mutations: Tyr145Asp and Asn193Asp. Finally, 10 ns MD simulations of the CYP51 wild-type protein and of the two of its mutants, Tyr145Asp and Asn193Asp were performed following a 5 ns equilibration period of each system. Lanosterol is axially coordinated to heme iron with the methyl carbon C30, which is the lanosterol center of oxidation. Tyr145, which was mutated, is located in the active site and is in close proximity to lanosterol, while Asn193 is located on the protein distal surface opposite and far from the active site (Figure S3). Results show only modest response of binding properties between lanosterol and wild type CYP51 and its mutants (Figure S2A). On average, lanosterol in the wild-type and Asn193Asp mutant is bound stronger to the protein compared to Tyr145Asp mutant, which is indicated by lower interaction energy. As a control parameter we choose the distance between the heme iron and C30 atom of the lanosterol (Figure S2B). The distance, which is measured every 1 ps, fluctuates in the three systems in the range of 3 to 6 Å and on average, turns out to be the shortest for the wild type and the longest for the mutant Tyr145Asp. C30 atom from lanosterol is thus in the Tyr145Asp mutant more distant from heme compared to the wild type.

We next investigated the bonds between heme and Tyr145 or the Asp145 mutant. It was shown previously that Tyr145 forms an H-bond with heme ring D propionate [31] which was confirmed by our hydrogen-bonding analysis over entire 10ns long MD trajectory. However, Asp145 is too distant to the heme to form a hydrogen bond (Figure 3). Substitution of a polar uncharged with a negatively charged amino acid influences the electrostatic properties of the proximal protein surface (Figure S3C) which may affect the binding to the obligatory redox partner POR. The interactions between POR and the different CYPs determine that the binding site for POR is located on the proximal surface of CYPs, however they also indicate that ionic-charge clusters mediate the CYP-POR interactions[42], [43], [44]. Electrostatic potential surface of POR is mostly negative [45]. The CYP51 protein proximal surface (Figure S3C) becomes more negative due to mutation, which might critically affect binding of POR [43], [46]. More negative is also the distal surface of the mutant Asn193Asp protein resulting from c.595T>C mutation (Figure S3D), but this residue is likely not linked to interactions with POR.

## Discussion

Investigating the risk factors of complex traits like preterm delivery and low birth weight require multiple techniques to better understand the genetic background and biochemical consequences that contribute to cholesterol levels in mother and the fetus. Herein we investigated the *CYP51A1* from cholesterol synthesis where the complete loss of function is embryo-lethal in the mouse [23]. Consequently, we expected that functional homozygous *CYP51A1* mutations would not be observed, which was indeed the case.

*CYP51A1* is a gene of low nucleotide variability with relatively few missense mutations [37]. To date there is a total of 340 *CYP51A1* SNPs (60 common variants found in ≥1% of samples) reported in the dbSNP 137 database among which 30 are rare missense mutations (9%) (not including our newly discovered variants). About 15 polymorphisms reside per 1 kb of *CYP51A1*, similar to what is seen for the regulatory gene of cholesterol synthesis *HMGCR* (16.5). Two other genes of cholesterol synthesis where mutations have already been associated with human disorders have a higher number of polymorphisms per 1 kb: *DHCR24* has 21.0 and *DHCR7* 26.2, where many associate with Smith-Lemli-Opitz syndrome [47]. For comparison, a highly polymorphic *CYP2D6* which is not from cholesterol synthesis but is a cytochrome P450 from the xenobiotic degradation pathway contains 75.1 polymorphisms per 1 kb, where 28% are missense [48]. *CYP2D6* thus contains over 3-fold more missense mutations compared to the essential *CYP51A1.* According to predictions (Table S3), seven to nine of the previously reported *CYP51A1* missense mutations are probably damaging (scoring 0.961 to 1.000), three are possibly damaging (0.647 to 0.858) while the remaining 20 are likely not affecting protein stability (<0.400).

Since missense mutations of *CYP51A1* are all heterozygous and rare, it is possible that mutation in a single allele negatively influences the CYP51 protein structure and lowers the enzyme activity which might become insufficient for normal cholesterol synthesis. The prediction software estimates only impact on protein stability, thus we investigated impact of all novel substitutions, found in preterm neonates, on protein structure performing molecular modeling. For example, the *CYP51A1* missense mutation leading to substitution of Tyr145Asp in the CYP51 substrate recognition site was predicted to be probably damaging. It is very rare and is so far associated with a single case of preterm baby and his mother. Previous experimental substitution of Tyr145 with His did not demonstrate an effect on the substrate turnover [41]. In the natural mutant the Tyr is changed to Asp resulting in lack of hydrogen bonding between Asp145 and the heme ring D propionate (Figure 3).

Our analysis shows that the investigated *CYP51A1* novel variants have little contribution to prematurity due to very low frequency, but they are of potentially big effect size, which we investigated with molecular modeling. They all seem to be very rare, possibly due to damaging effects already in the heterozygous state. Also the investigated common *CYP51A1* variant rs6465348 did not show a direct association with prematurity by family based approach. However, the recessive (C) allele of rs6465348 was significantly less frequent in neonates born small for gestational age. There are a number of risks for adverse outcome arising from the decreased birth weight, including oxygen saturation [49], higher morbidity [50] and parenteral nutrition associated cholestasis [51]. Children born with SGA weight have to be closely monitored and may require additional health care irrespective of prematurity. It is thus important to know that *CYP51A1* genotype is among factors that may contribute to normal growth of the fetus.

In addition to the genotype of the baby, maternal factors may also contribute to the multifactorial preterm delivery and low birth weight. Maternal cholesterol levels during the pregnancy have been a subject of recent studies [52], [53], [54], [55]. They show that low lipid values (total cholesterol TC, HDL-c and LDL-c) modestly associate with medically indicated PTD (in women whose labor was induced or who were given cesarean sections before the onset of labor, or premature rupture of membranes), whereas high blood lipids associate with spontaneous PTD [11]. We identified association of *CYP51A1* SNP rs6465348 CC genotype with lower total cholesterol and LDL-c during the second trimester of pregnancy. The link between maternal cholesterol levels during both early and late pregnancy and adverse birth outcomes is not conclusive [52], [56], [57]. Our data indicate the relationship between the genotype of the mother and cholesterol levels during the pregnancy.

## Concluding Remarks

Due to the critical need for cholesterol synthesis and the evolutionary conserved role of the CYP51 protein, opportunities to detect homozygous functional *CYP51A1* polymorphisms in humans are minor. *CYP51A1* has thus so far not been directly linked to human diseases. High throughput sequencing (the 1000 genome database, NHLBI-ESP) shed a light on population variability of essential genes such as *CYP51A1. CYP51A1* is less polymorphic compared to non-essential genes from the same family (*CYP2D6* taken as example) and has relatively few missense mutations, all detected with very low minor allele frequency. One malfunctioning allele might lead to insufficient protein for normal function, as indicated also from studies in our *Cyp51* mouse model. The lack of a single *CYP51A1* allele found in preterm infant and its mother who gave preterm birth is important and novel, even if the variant shows very low frequency. Association of the rs6465348 with SGA and lower blood cholesterol and LDL cholesterol gives valuable information and suggests that *CYP51A1* gene may play an important role in pathologies of pregnancy even if it does not associate directly with PTD. We believe that single allele polymorphisms of essential genes have a much higher impact than previously anticipated, since rare (heterozygous) events might be missed by genome wide studies. A step forward in understanding the role of cholesterol linked genotypes is deep sequencing of these genes in patient groups with cholesterol-related multifactorial disorders.

## Acknowledgments

We thank the many families who participated in this study; Dr. Galina Lepesheva from Vanderbilt University School of Medicine for helpful discussion; members of Murray laboratory especially Julia Tanguay, Tamara Busch and Allison Momany for technical assistance; Susie McConnell, Erin Brothers-Smith, and Nancy Davin for administrative assistance.

## Supporting Information

Figure S1 Position of lanosterol in the active site of human CYP51 after docking. Three poses of lanosterol molecule with the lowest GlideScore values are shown in blue, yellow and purple. The image was designed using VMD.
(TIF)

Figure S2 **A** Interaction energies between lanosterol and CYP51 of the wild-type (red), Tyr145Asp (green) and Asn193Asp (blue). The interactions present binding properties of wild-type protein and mutants, higher values for Tyr145Asp mutant indicates modestly decreased binding of lanosterol. The error bars are showing the local standard deviation of binding energies. **B** Interactomic distances between the heme Fe and C30 atom of the lanosterol of the wild-type (red), Tyr145Asp (green) and Asn193Asp (blue).
(TIF)

Figure S3 The electrostatic potential surface of CYP51. Panels **A** and **B** show the front (proximal to the active site) and rear (distal to the active site) surface of the wild-type CYP51 displaying the residues Tyr145 in green and Asn193 in purple, respectively. Panels **C** (front) and **D** (rear) show the mutants Tyr145Asp in green and Asn193Asp in purple, respectively. Surface electrostatic potentials are shown scaled with the color intensity: positive potential in blue and negative potential in red.
(TIF)

Table S1 Primer sequences generated with Primer3 software and amplicons sizes.
(DOCX)

Table S2 Minor allele frequencies, and test for Hardy-Weinberg Equilibrium performed with chi-square test and exact test p-values for known *CYP51A1* variants that have been identified by sequencing.
(DOCX)

Table S3 PolyPhen-2 and SIFT prediction of functional mutations in CYP51 NP_000777.1 reported in dbSNP 137 with minor allele frequencies calculated in all samples available in EVS (Exome Variant Server NHLBI Exome Sequencing Project).
(DOCX)

Table S4 Lanosterol poses produced after docking and corresponding GlideScore values.
(DOCX)
