# Supplementary figures and images for: Polymorphisms of CYP51A1 from Cholesterol Synthesis: Associations with Birth Weight and Maternal Lipid Levels and Impact on CYP51 Protein Structure
Source: PLoS One. 2013 Dec 17;8(12):e82554. doi: 10.1371/journal.pone.0082554 (PMC3866192; doi:10.1371/journal.pone.0082554)

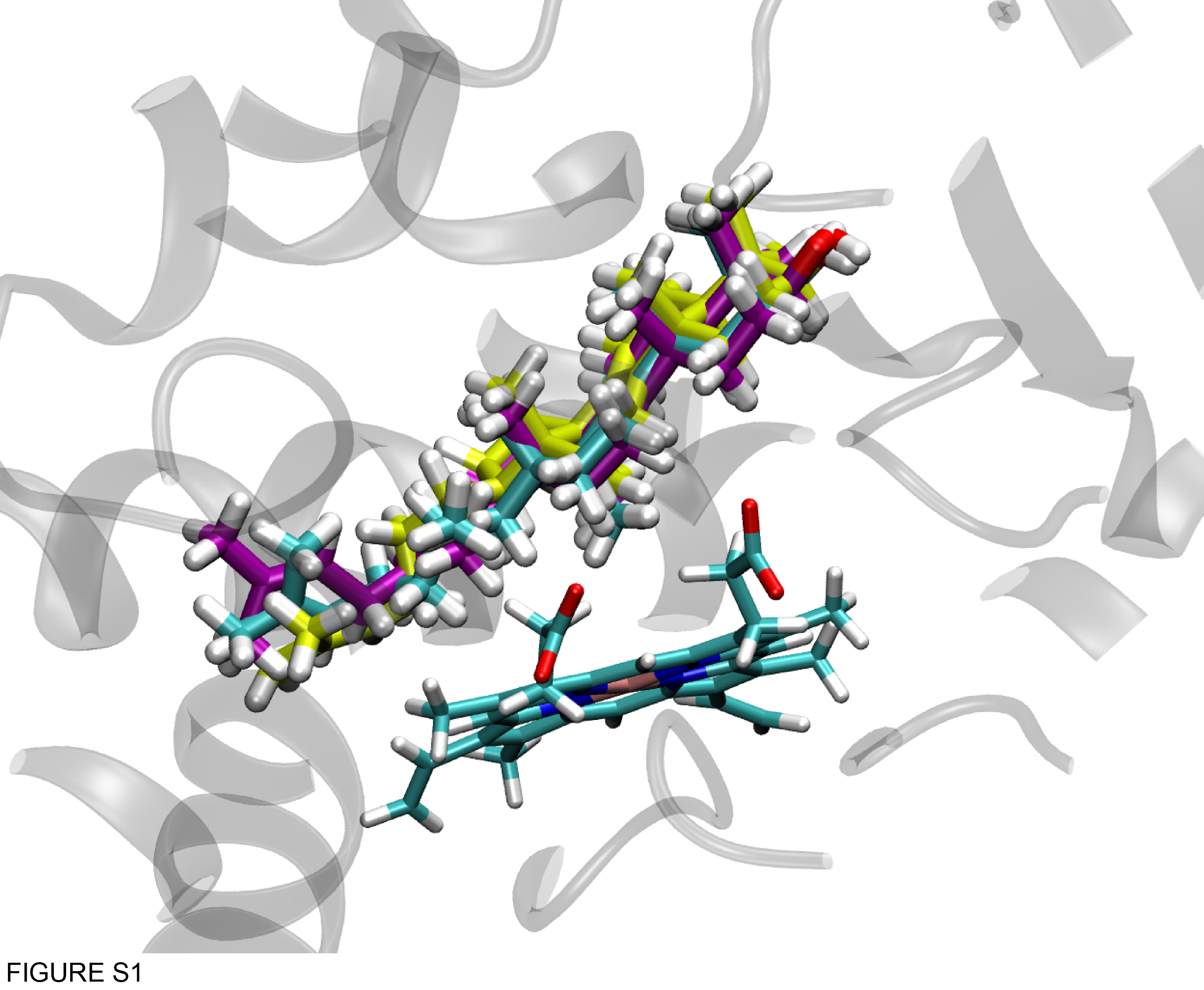

Supplement: Figure S1 — Position of lanosterol in the active site of human CYP51 after docking. Three poses of lanosterol molecule with the lowest GlideScore values are shown in blue, yellow and purple. The image was designed using VMD. (TIF) [file pone.0082554.s001.tif]

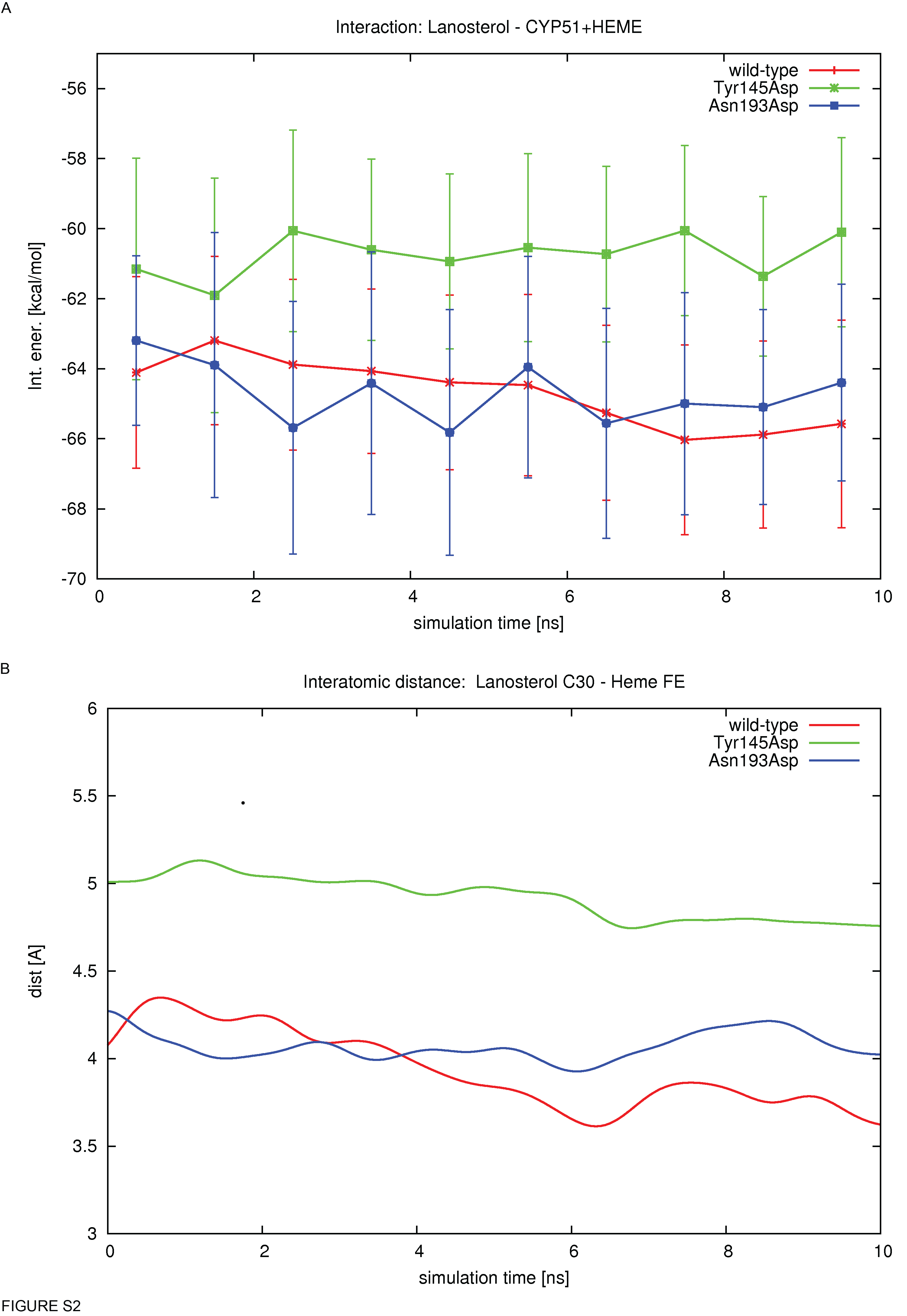

Supplement: Figure S2 — A Interaction energies between lanosterol and CYP51 of the wild-type (red), Tyr145Asp (green) and Asn193Asp (blue). The interactions present binding properties of wild-type protein and mutants, higher values for Tyr145Asp mutant indicates modestly decreased binding of lanosterol. The error bars are showing the local standard deviation of binding energies. B Interactomic distances between the heme Fe and C30 atom of the lanosterol of the wild-type (red), Tyr145Asp (green) and Asn193Asp (blue). (TIF) [file pone.0082554.s002.tif]

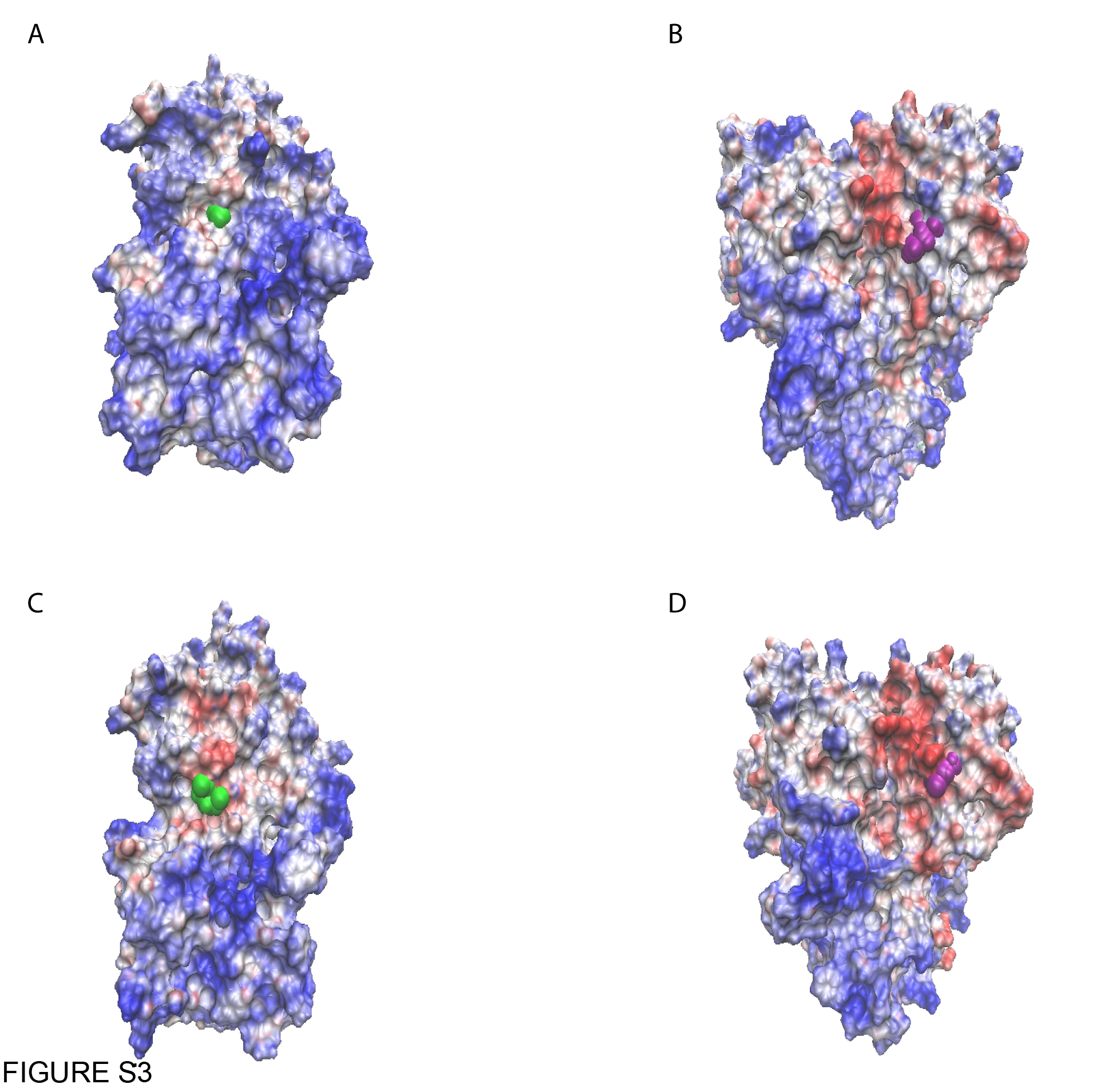

Supplement: Figure S3 — The electrostatic potential surface of CYP51. Panels A and B show the front (proximal to the active site) and rear (distal to the active site) surface of the wild-type CYP51 displaying the residues Tyr145 in green and Asn193 in purple, respectively. Panels C (front) and D (rear) show the mutants Tyr145Asp in green and Asn193Asp in purple, respectively. Surface electrostatic potentials are shown scaled with the color intensity: positive potential in blue and negative potential in red. (TIF) [file pone.0082554.s003.tif]
