# Supplementary material for: Polymorphisms of CYP51A1 from Cholesterol Synthesis: Associations with Birth Weight and Maternal Lipid Levels and Impact on CYP51 Protein Structure
Source: PLoS One. 2013 Dec 17;8(12):e82554. doi: 10.1371/journal.pone.0082554 (PMC3866192; doi:10.1371/journal.pone.0082554)
Supplement: Table S1 — Primer sequences generated with Primer3 software and amplicons sizes. (DOCX) [file pone.0082554.s004.docx]

**Table S1.** Primer sequences generated with Primer3 software and amplicons sizes.

| *CYP51*  amplicon number | Forward primers | Reverse primers | amplicon size bp |
| --- | --- | --- | --- |
|  | 5`-3` sequence | 5`-3` sequence |  |
| 1 | CGATTCTCAGGGATTGATCG | ACGACCCCGTCTAGGATCAG | 807 |
| 2 | TGGTCATGAAACGAAACTGG | AAAGTTTAAAAAGCACTTCTCTAGTTG | 236 |
| 3 | TTTAAAAAGTTACTGTCGTAGTGTTGC | CATGCCTGGCTGTTTCTACA | 347 |
| 4 | GACTCTTAAAATGTAAATGCCCAAA | CATTACATAACCCTCCCATAAATC | 406 |
| 5 | TTCACTTTTTGGAAATAGTAATGAGAA | GGCAAAGCATACCAACACTACA | 294 |
| 6 | TCGGTCCTGGTTCTCAAAAT | TGTGTAATTTCCTGCTTCAGCTT | 249 |
| 7 | GCATGAAATGTATCTGCCAAAA | AAGGCAGCAATGAAACTGAAA | 282 |
| 8 | AGCTGGAATGCAAGCTGTCT | CCACCACACCCACCTATTTC | 274 |
| 9 | TGATCTGATTATTTGTCAGCACATT | TGTTATCAGAACAAACATTTCCAA | 277 |
| 10 | ACCCCAGGCTAAAAAGGATG | TGGATTCCCTAACTCTGCTCA | 1814 |
