# Supplementary material for: Polymorphisms of CYP51A1 from Cholesterol Synthesis: Associations with Birth Weight and Maternal Lipid Levels and Impact on CYP51 Protein Structure
Source: PLoS One. 2013 Dec 17;8(12):e82554. doi: 10.1371/journal.pone.0082554 (PMC3866192; doi:10.1371/journal.pone.0082554)
Supplement: Table S2 — Minor allele frequencies, and test for Hardy-Weinberg Equilibrium performed with chi-square test and exact test p-values for known CYP51A1 variants that have been identified by sequencing. (DOCX) [file pone.0082554.s005.docx]

**Table S2** Minor allele frequencies, and test for Hardy-Weinberg Equilibrium performed with chi-square test and exact test p-values for known *CYP51A1* variants that have been identified by sequencing

| rs number | MAF dbSNP | MAF neonates | χ2 p-value  neonates | Exact test  p-value | MAF mothers | χ2 p-value  mothers | Exact test  p-value |
| --- | --- | --- | --- | --- | --- | --- | --- |
| rs117814311 | 0.008 | 0.015 | 0.999 | 1.000 | 0.008 | 0.993 | 1.000 |
| rs189739058 | 0.001 | 0.003 | 0.999 | 1.000 | 0.000 | N/A | N/A |
| rs142544033 | 0.001 | 0.000 | N/A | N/A | 0.003 | 0.999 | 1.000 |
| rs147205401 | 0.003 | 0.006 | 0.997 | 1.000 | 0.014 | 0.985 | 1.000 |
| rs57218044 | 0.032 | 0.003 | 0.999 | 1.000 | 0.000 | N/A | N/A |
| rs184213287 | 0.002 | 0.003 | 0.999 | 1.000 | 0.000 | N/A | N/A |
| rs59683852 | 0.005 | 0.003 | 0.999 | 1.000 | 0.000 | N/A | N/A |
| rs7797834 | 0.355 | 0.418 | 0.979 | 0.979 | 0.360 | 0.337 | 0.556 |
| rs7793861 | 0.358 | 0.413 | 0.985 | 0.979 | 0.429 | 0.882 | 0.919 |
| rs6465348 | 0.353 | 0.416 | 0.975 | 0.979 | 0.420 | 0.889 | 0.961 |
| rs12673910 | 0.180 | 0.119 | 0.936 | 0.935 | 0.061 | 0.166 | 0.790 |
| rs1135217 | 0.358 | 0.384 | 0.797 | 0.917 | 0.363 | 0.096 | 0.346 |
