# Supplementary material for: Polymorphisms of CYP51A1 from Cholesterol Synthesis: Associations with Birth Weight and Maternal Lipid Levels and Impact on CYP51 Protein Structure
Source: PLoS One. 2013 Dec 17;8(12):e82554. doi: 10.1371/journal.pone.0082554 (PMC3866192; doi:10.1371/journal.pone.0082554)
Supplement: Table S3 — PolyPhen-2 and SIFT prediction of functional mutations in CYP51 NP_000777.1 reported in dbSNP 137 with minor allele frequencies calculated in all samples available in EVS (Exome Variant Server NHLBI Exome Sequencing Project). (DOCX) [file pone.0082554.s006.docx]

**Table S3** PolyPhen-2 and SIFT prediction of functional mutations in *CYP51* NP_000777.1 reported in dbSNP 137 with minor allele frequencies calculated in all samples available in EVS (Exome Variant Server NHLBI Exome Sequencing Project)

|  | rs number | MAF in EVS | NP_000777.1 AA position | PolyPhen-2 prediction (score) | SIFT prediction (score) |
| --- | --- | --- | --- | --- | --- |
|  | rs144033175 | Not found | p.Met7Leu | Benign (0.000) | Damaging (0.00)^a^ |
|  | rs138006785 | 0.000386 | p.Ala15Val | Benign (0.000) | Tolerated (0.55) |
|  | rs2229188 | Not found | p.Val19Ala | Benign (0.034) | Tolerated (0.27) |
|  | rs141141306 | 0.000077 | p.Arg51His | Benign (0.010) | Tolerated (0.57) |
|  | rs148197561 | 0.000308 | p.Ile74Val | Benign (0.001) | Tolerated (0.22) |
|  | rs138205508 | 0.001692 | p.Ile88Val | Benign (0.003) | Tolerated (0.52) |
|  | rs149112643 | 0.000154 | p.Ile88Thr | Benign (0.023) | Tolerated (0.10) |
|  | rs146474563 | 0.000077 | p.Met106Val | Benign (0.026) | Tolerated (0.14) |
|  | rs140356336 | 0.000077 | p.Arg139His | Benign (0.197) | Tolerated (0.70) |
|  | rs146573154 | 0.000077 | p.Pro156Leu | Benign (0.057) | Tolerated (0.26) |
|  | rs144068591 | Not found | p.Met164Leu | Benign (0.103) | Tolerated (0.23) |
|  | rs139239552 | 0.000077 | p.Ile171Val | Benign (0.002) | Tolerated (0.65) |
|  | rs151249652 | 0.000154 | p.Tyr188Cys | Probably Damaging (1.000) | Damaging (0.00) |
|  | rs142437178 | 0.000077 | p.lys228Gln | Benign (0.001) | Tolerated (0.50) |
|  | rs200921006 | Not found | p.Arg258Cys | Probably Damaging (0.999) | Damaging (0.00) |
|  | rs141009880 | 0.000154 | p.Ile274Thr | Possibly Damaging (0.722) | Damaging (0.02) |
|  | rs140702410 | Not found | p.Arg277Leu | Probably Damaging (0.999) | Damaging (0.00) |
|  | rs146408738 | Not found | p.Asp285Tyr | Probably Damaging (0.967) | Damaging (0.01) |
|  | rs143968550 | 0.000077 | p.Ala308Val | Probably Damaging (0.961) | Damaging (0.00) |
|  | rs140118347 | 0.000077 | p.Ala334Ser | Benign (0.363) | Damaging (0.04) |
|  | rs184629573 | Not found | p.Lys341Glu | Benign (0.000) | Tolerated (1.00) |
|  | rs138355676 | 0.000308 | p.Arg371Leu | Benign (0.009) | Tolerated (0.68) |
|  | rs150090274 | 0.000077 | p.Ile383Val | Benign (0.03) | Tolerated (0.07) |
|  | rs139028687 | Not found | p.Thr400Ala | Benign (0.048) | Tolerated (0.26) |
|  | rs138109473 | 0.000077 | p.Arg431His | Probably Damaging (1.000) | Damaging (0.00) |
|  | rs59683852 | 0.004383 | p.Pro437Ser | Benign (0.285) | Tolerated (0.73) |
|  | rs55756240 | Not found | p.Lys466Arg | Probably Damaging ( 0.961) | Tolerated (0.60) |
|  | rs145784242 | 0.000077 | p.Ile481Thr | Possibly Damaging (0.647) | Tolerated (0.16) |
|  | rs140738858 | Not found | p.Pro500Ser | Possibly Damaging (0.858) | Tolerated (0.16) |
|  | rs202233563 | Not found | p.Ser508Thr | Benign (0.000) | Tolerated (0.22) |

^a^Low confidence
