# Supplementary material for: Polymorphisms of CYP51A1 from Cholesterol Synthesis: Associations with Birth Weight and Maternal Lipid Levels and Impact on CYP51 Protein Structure
Source: PLoS One. 2013 Dec 17;8(12):e82554. doi: 10.1371/journal.pone.0082554 (PMC3866192; doi:10.1371/journal.pone.0082554)
Supplement: Table S4 — Lanosterol poses produced after docking and corresponding GlideScore values. (DOCX) [file pone.0082554.s007.docx]

**Table S4** Lanosterol poses produced after docking and corresponding GlideScore values.

| Lanosterol  pose number | GlideScore |
| --- | --- |
| 1 | -8.668 |
| 2 | -8.644 |
| 3 | -8.605 |
| 4 | -8.562 |
| 5 | -8.452 |
| 6 | -8.202 |
| 7 | -8.065 |
| 8 | -7.794 |
| 9 | -7.293 |
| 10 | -7.100 |
